# Enhancing the Mechanical Properties of Glass-Ionomer Dental Cements: A Review

**DOI:** 10.3390/ma13112510

**Published:** 2020-05-31

**Authors:** John W. Nicholson, Sharanbir K. Sidhu, Beata Czarnecka

**Affiliations:** 1 Dental Materials Unit, Bart’s and the London Institute of Dentistry, Queen Mary University of London, Mile End Road, London E1 4NS, UK; 2 Bluefield Centre for Biomaterials, 67-68 Hatton Garden, London EC1N 8JY, UK; 3 Centre for Oral Bioengineering, Institute of Dentistry, Bart’s & The London School of Medicine and Dentistry, Queen Mary University of London, Turner Street, London E1 2AD, UK; s.k.sidhu@qmul.ac.uk; 4 Department of Biomaterials and Experimental Dentistry, Poznań University of Medical Sciences, ul. Bukowska 70, 60-812 Poznań, Poland; czarnecka@ump.edu.pl

**Keywords:** glass-ionomer cements, resin-modified, fibre, reinforcement, nanoparticles, testing, strength

## Abstract

This paper reviews the strategies that have been reported in the literature to attempt to reinforce glass-ionomer dental cements, both conventional and resin-modified. These cements are widely used in current clinical practice, but their use is limited to regions where loading is not high. Reinforcement might extend these applications, particularly to the posterior dentition. A variety of strategies have been identified, including the use of fibres, nanoparticles, and larger particle additives. One problem revealed by the literature survey is the limited extent to which researchers have used International Standard test methods. This makes comparison of results very difficult. However, it does seem possible to draw conclusions from this substantial body of work and these are (1) that powders with conventional particle sizes do not reinforce glass-ionomer cements, (2) certain fibres and certain nanoparticles give distinct improvements in strength, and (3) in the case of the nanoparticles these improvements are associated with differences in the morphology of the cement matrix, in particular, a reduction in the porosity. Despite these improvements, none of the developments has yet been translated into clinical use.

## 1. Introduction

Glass-ionomer cements (GICs) are widely used in clinical dentistry with uses including full restorations, liners and bases, luting agents, fissure sealants, and adhesives for orthodontic brackets [1]. Their properties are generally attractive for these applications and include biocompatibility towards tooth tissue [2], the ability to release fluoride [3,4], and adhesion to the tooth surface [5,6]. They also match the natural tooth tissue in terms of coefficient of thermal expansion [7].

Glass-ionomers are made from special basic glass powders that are either calcium or strontium alumino-fluoro-silicate with additions of phosphate, typically AlPO_4_, and also sodium salts [8]. These are able to react with a solution of polymeric water-soluble acid to form salts that effectively crosslink the polymer chain and cause the material to harden [1,9,10,11]. The most widely used polymer is polyacrylic acid but cements can also be prepared with acrylic/maleic or acrylic/itaconic copolymer or the copolymer of 2-methylene butanedioic acid with propenoic acid [11]. Commercial formulations often include some of the polymeric acid mixed as a dry powder with the glass. This effectively increases the concentration of the acidic polymer in the final cement without making the liquid to be too viscous to mix. Large amounts of polymeric acid make the resulting cement strong [10], a feature which is necessary for clinical durability.

As well as the formation of carboxylate crosslinks, there is also a secondary setting reaction of certain inorganic species released from the glass [12]. The most important of these components appears to be the phosphate, since without phosphate, ionomer glasses do not give insoluble cements with simple monomeric acids [13]. In glass-ionomer cements, the gradual formation of a modified network from these inorganic species has been proposed as being partly responsible for the changes that take place as the glass-ionomer matures. These changes include decreased plasticity, greater compressive strength, and improved translucency [14].

Glass-ionomer cements of this type were as originally reported in the early 1970s [15] and are now known as conventional glass-ionomers. This is to distinguish them from the resin-modified glass-ionomers (RMGICs), materials first described about 20 years later [16,17]. RMGICs contain the same components as conventional glass-ionomers, namely basic glass powder, water, and polyacid. They also contain a monomer component and associated initiator system. The monomer is usually 2- hydroxyethyl methacrylate, HEMA, and the initiator is the light-sensitive substance camphorquinone [10]. Resin-modified glass-ionomers set by a combination of neutralization (acid-base reaction) and addition polymerization. The set material has a complicated structure based on the blended polymer and polysalt components that arise from these two reactions [18]. Competition between these two network-forming systems results in a delicate balance between them [19], meaning that delay in applying light to initiate the photochemical polymerization reaction can alter the properties of the resulting material [19,20]. Hence, it is important that the manufacturer’s recommendations on the duration and timing of the light-cure step are followed closely so that the set material has optimal properties [19].

RMGICs can release the monomer HEMA in varying amounts early in the life of the restoration [21]. The extent of light-curing is an important factor in controlling the amount released, and release also varies with depth of the restoration, because lower layers receive less light intensity and so polymerize to a smaller extent. This, in turn, leaves more unreacted HEMA monomer in the structure, and this free HEMA is able to leach from the cement into the adjacent tooth tissue. HEMA can diffuse through human dentine [22] and is cytotoxic to the cells of the pulp [23,24].

HEMA from resin-modified glass-ionomers can also be the cause of problems for dental personnel. It is a contact allergen and is volatile, so it can be inhaled [24], where it can cause respiratory problems. Dentists should work in a well-ventilated area and make sure that no vapor is inhaled [25]. They are also advised to light-cure left-over material before disposing of it.

Resin-modified glass-ionomers have mainly the same clinical applications as conventional glass-ionomers [26,27], though they are not recommended for the atraumatic restorative treatment (ART) technique. They can be used in Class I, Class II, and Class III restorations, all mainly in the primary dentition, Class V restorations, and also as liners and bases [28]. Their other uses include as fissure sealants [28] and as bonding agents for orthodontic brackets [29].

Despite the many years of development of these materials and the corresponding amount of experience in using them clinically, these materials still have limitations [1]. These limitations arise from the mechanical properties of the set cement. In particular, conventional glass-ionomers are brittle materials with compressive strengths in the range 150–220 MPa and resin-modified glass-ionomers, while tougher and with better flexural strength, have comparable compressive properties. As a result, both types of material have similar limitations for clinical use [1,10]. There have been numerous attempts to reinforce these materials, some of which have been used in clinical materials and others of which still remain experimental. This paper reviews the various approaches to reinforcement that have been reported in the literature for these materials, covering both conventional and resin-modified glass-ionomers.

## 2. Mechanical Properties of Conventional and Resin-Modified Glass-Ionomer Cements

Before considering the topic of reinforcement, it is appropriate to consider the mechanical properties of glass-ionomer cements of both types. This is important not only to establish the baseline from which reinforcement has to be achieved, but also because some authors refer to the latter group of glass-ionomers as “resin-reinforced”. This is not the preferred term, and the correct term is resin-modified, as first proposed in the mid-1990s [30], and widely used throughout the literature [31].

It is difficult to compare these classes of material, because the relevant international standards specify different strengths [32]. Conventional glass-ionomer cements are tested for compressive strength and have a minimum requirement of 100 MPa for restorative use in patients. On the other hand, resin-modified glass-ionomers are specified to be tested for flexural strength and should have a minimum strength of 20 MPa for clinical use [33].

A few published studies have reported compressive strength values for resin-modified glass-ionomers and flexural strength values for conventional glass-ionomers in attempts to compare the materials. Unfortunately, the reported values vary widely, and make it impossible to draw any reliable conclusions, despite the claims of the individual studies. For example, the conventional glass-ionomer Fuji IX (GC, Tokyo, Japan) in its hand-mixed form has been reported to have a compressive strength of 83.6 MPa at 24 h in one study [34] and 350.87 MPa in another [35]. The former study then concludes that resin-modified glass-ionomers are stronger in all test modes, but with such a low reported compressive strength for the conventional cement, how can the data be trusted? This is not to question the ability or skill of the researchers: These are difficult materials to mix [36] and there are many factors to be controlled when preparing specimens for testing. Doing so reliably is extremely challenging.

Other low values of compressive strength have been reported for conventional glass-ionomers. For example, the material Ionofil Molar (VOCO, Cuxhaven, Germany) was reported to have a compressive strength of 78.78 MPa, well below that of the resin-modified materials Vitremer (3M-ESPE, Seefeld, Germany), which had a compressive strength of 169.50 MPa [37]. While it is interesting to observe how high the compressive strength of a resin-modified glass-ionomer can be, the value for the conventional glass-ionomer must be questioned. It is lower than that recommended by the appropriate international standard [32], an improbable outcome for a material from a reputable manufacturer.

Despite these variations in reported values in individual papers, there does seem to be an overall consensus that RMGICs are superior to conventional materials in their diametral tensile strength [38,39] and in their flexural strength [39]. In a recent study, the superiority of the toughness and flexural properties was shown to be consistent across a range of brands [40]. For example, the fracture toughness at 24 h of a number of commercial conventional glass-ionomers ranged from 0.18 to 0.30 MPa m^½^ compared with 0.49–0.67 MPa m^½^ for a similar set of commercial RMGICs. For the same materials, flexural strength at 24 h ranged from 18 to 34 MPa for the conventional glass-ionomers compared with 49–76 MPa for the RMGICs [40]. These differences are attributable to the presence of the polymerized resin component, which toughens the cement and improves its ability to withstand loading in flexure.

## 3. Comparison with Tooth Materials

Glass-ionomers of both types are used to repair teeth that have been damaged, mainly by caries. In the context of considering how to improve the mechanical properties of glass-ionomers, it is appropriate to consider the mechanical properties of the natural tooth.

Teeth consist of two main types of material, the dentine and the enamel, and they have very different mechanical properties. Both are anisotropic [41], but whereas dentine is relatively tough, enamel is brittle. As a consequence of their geometry, the main properties of these tissues that have been evaluated are those that can be determined by nano-indentation [42,43], namely hardness, modulus, and fracture toughness. Values of these properties are shown Table 1.

In the case of enamel, mechanical properties vary widely across the enamel layer. For example, external enamel has a fracture toughness of around 0.67 ± 0.12 MPa m^½^, whereas internal enamel has values of up to 3.93 MPa m^½^. This means that the internal enamel has a much greater crack resistance [48] and this arises because the detailed composition and arrangement of the enamel rods differ in these varied locations.

The dentine has more consistent properties throughout its structure, though even this material varies close to the dentino-enamel junction, DEJ. Dentine is a viscoelastic material [52], which means it is much tougher than enamel, and more resistant to fracture.

The DEJ is the region in which the dentine and the enamel are joined [42]. Although enamel and dentine have very different properties, there is a transition in properties of both in the regions close to the DEJ [53]. Although the DEJ is itself brittle, as are the two structures adjacent to it, the overall effect of its presence is that cracks do not propagate through it. This has the effect of making the tooth mechanically strong.

The exact value of strength is difficult to measure, due to the difficulty of preparing uniform test specimens, or even of determining cross-sectional areas of loaded teeth. One paper dealt with the problem by simplifying the experimental data and recording the load at failure of teeth as they underwent fracture [54]. Teeth were extracted and tested in compression in a universal testing machine, either as obtained, with a cut cavity, or with a cavity filled with composite resin (10 samples per condition). Results are shown in Table 2, and they demonstrate that the mere act of cutting a cavity weakens the tooth significantly. Conversely, repairing the cavity goes a long way to restoring the strength of the tooth, even though the final value was still below that of the natural tooth.

There is evidence that these values change throughout the lifetime of individuals. The teeth of the elderly are more susceptible to fracture than those of the young [55], though whether this is because they become more brittle with age, suffer fatigue, or simply become weaker, is not clear.

Given the differences in mechanical properties of enamel and dentine, and the variations in the mechanical properties of these tissues with precise location within the tooth and also with the age of the patient, the suggestion by some researchers that glass-ionomers should match the properties of natural teeth is clearly not practicable. Instead, improving the properties of glass-ionomers to enhance their durability should be the goal. Probably the key requirement is to improve the flexural strength and Young’s modulus, because these properties correlate with wear and, hence, clinical durability [37,56], though other properties, such as hardness, are also important in influencing durability. Steps that have been studied as possible means of improving the mechanical properties of glass-ionomers are considered in the following sections of this review.

## 4. Metal Reinforcement

Metal reinforcement has been applied to conventional glass-ionomers used in the dental clinic for many years [57]. The original concept was to develop a glass-ionomer cement to replace amalgams. Two approaches have been used, namely the incorporation of metal powders, mainly silver-tin alloy [58], and the incorporation of silver-tin alloy fused with the ionomer glass powder, a so-called cermet [59].

The earliest report of metal powders for reinforcement of glass-ionomer cements was in a patent by Wilson and Sced granted in 1980 [60]. They described the addition of powders of aluminum, chromium, nickel-aluminum alloy, and silver-tin alloy, all of which were of relatively large particle size, i.e., not nanoparticles. All were claimed to improve the flexural strength [60,61]. Of these, the silver-tin alloy gave the greatest increase, raising the reported value to 40 MPa from a value of 10 MPa for the parent cement.

Silver-tin alloy of the type typically used in amalgams incorporated into a glass-ionomer was marketed as a material known as “Miracle Mix” by the GC company from 1983 [61]. It is still on the market as of the start of 2020, and is a useful material for certain niche applications [62]. Results with it in terms of reinforcement have been mixed, with some authors claiming it is stronger than the typical unmodified glass-ionomer cement Fuji IX (GC, Tokyo, Japan) [63], and others claiming it is weaker [64]. The overall conclusion is that there is no clear improvement in strength [57], but this does not seem to matter, because its continued use does not seem to be because of its extra strength, but because it is radio-opaque.

The alternative approach, namely of the silver alloy being fused to the glass to form a cermet (from the words *cer*amic and *met*al), involves a change at the manufacturing stage. Like the inclusion of metal powder, the result is a cement with compromised aesthetics but improved radio-opacity. The cermet system was commercialized in 1986, when the material Chelon Silver was launched by the ESPE GmbH company [61]. As with Miracle Mix, this material is still on the market at the time of writing (April 2020).

Early studies on strength were carried out, with the cermet cement being compared with the conventional glass-ionomer cement Chelon Fil (ESPE, Seefeld, Germany) Williams et al. reported that the cermet cement had lower strengths than the conventional glass-ionomer [65]. For example, diametral tensile strength was found to be 13 ± 2 MPa compared with 19 ± 3 MPa for Chelon Fil. Walls et al. [66] confirmed these findings for flexural strength, using a three-point bending test, which showed Chelon Silver to have a strength of 29 ± 7 MPa and Chelon Fil to have a strength of 45 ± 5 MPa. The overall conclusion from these and other studies is that cermet-based glass-ionomers are not reinforced at all and are actually weaker than cements made with conventional glass powders [57].

Another metal filler that has been considered for inclusion in glass-ionomers is stainless steel powder [67]. When compared with Miracle Mix, a cement containing finely divided stainless steel was shown to be stronger. For example, the diametral tensile strength was 23 ± 2 MPa compared with 11 ± 2 for Miracle Mix. However, despite this superiority, it is Miracle Mix that has been on the market since 1983, rather than a formulation containing stainless steel powder.

## 5. Fibre Reinforcement

The possibility of reinforcing glass-ionomer cements with fibres was also mentioned in the 1980 patent of Sced and Wilson [58]. They used both carbon and alumina fibres in a now-obsolete commercial glass-ionomer called Chembond (Dentsply deTrey, Konstanz, Germany), and examined the effect of fibres on flexural strength. Fibres employed were less than 1000 μm long and ranged in diameter from 10 to 200 µm. They were incorporated at 25 volume %, and increased the flexural strength substantially. The control cement had a flexural strength of 10 MPa, but alumina fibres raised this to 44 MPa and carbon fibres raised it to 53 MPa. Similar improvements in flexural strength have been reported by other authors for these types of fibre in different glass-ionomer formulations [64]. Carbon fibres have been claimed to be superior because, unlike alumina fibres, they do not cause the cement to increase in brittleness [68]. On the other hand, they alter the appearance and compromise the aesthetics.

Reactive glass fibres based on ionomer glass formulations have also been used to reinforce glass-ionomer cements. These glasses have been based on either the SiO_2_-Al_2_O_3_-CaO-P_2_O_5_ [69,70] or the SiO_2_-Al_2_O_3_-CaF_2_-Na_3_AlF_6_ [71] systems. The concept behind the use of these glasses is that, in order for fibres to properly reinforce a material, there must be good interfacial coupling of the matrix to the fibre [57]. Deploying fibres made of basic ionomer glasses ensures that such good coupling occurs and that the fibres are genuinely able to reinforce the cement. In principle, optimal results are obtained when the fibres are aligned with the direction of the primary load. In practice, though, short fibres become dispersed in random directions during mixing, so that improvements in strength may be only slight.

Typical results for the use of reactive glass fibres are shown in Table 3, and these are based on the findings of Lohbauer et al. [71]. The critical fibre length had previously been calculated to be 546 µm, so the fibres used to reinforce the cements, which had a diameter of 26 µm, were prepared to be 600 µm in length. The cement itself was based on components prepared specially for the study, rather than a commercial material. When fibres were incorporated, the highest flexural strength was achieved with 20 volume % loading, as shown in Table 3.

Following fracture, specimens were examined by fractography, and it was shown that specimens failed with fibre pull-out. This showed that the presence of the fibres increased strength by increasing the work of fracture, causing a corresponding increase in the fracture toughness.

Other studies have confirmed the reinforcing effect of glass fibres, though not with reactive glasses. Instead, nonbasic glass fibres have been used and incorporated into glass-ionomer cement formulations. In one study, relatively low amounts (3 and 5 weight %) were added to a commercial cement (Medifill, Promedica, Germany) and found to raise the diametral tensile strength from 7.49 (± 1.5) MPa to 9.15 (± 1.35) MPa at 3 weight % loading and 11.86 (± 2.27) MPa at 5 weight % loading [72].

Resin-modified glass-ionomers, too, have been reinforced by adding glass fibres. In this case, too, the fibres were prepared from nonreactive glasses [73], with both hollow and solid short length fibres being added at two different loadings, namely 5 and 10 weight %. A conventional and a resin-modified glass-ionomer were used as parent cements in the study. Results for the hollow-fibre modifications are shown in Table 4 and from these it can be seen that compressive strength was generally unaffected by the inclusion of these fibres, but that flexural strength and fracture toughness increased significantly. Both the conventional and the resin-modified glass-ionomer materials were substantially toughened by adding these fibres, and would be expected to show superior wear and durability if used clinically in patients.

Other fibres have also been used to reinforce glass-ionomers with some success. One material used has been basalt in fibre form [74]. Basalt is a mixture of the silicate minerals plagioclase, pyroxene, and olivine, and it can be readily formed into fibres. Technical uses for these fibres include the aerospace and automotive industries, where it is used a fireproof textile [75]. It has also been used to reinforce organic polymers, such as epoxy systems [76].

Basalt fibres were included in the conventional glass-ionomer Fuji IX in a study, with levels up to 7 weight % being used [74]. Early strength was found to increase significantly, but there was evidence that the interaction between the fibres and the matrix was weak, which suggested that the properties were likely to decline over the long term. Experimental studies confirmed this suggestion, as both flexural strength and flexural modulus of the fibre-reinforced cements declined over one month and became the same as the control material. No further reports have appeared on this material as a reinforcing fibre in glass-ionomers.

Cellulose microfibres have been studied as possible reinforcing agents for conventional glass-ionomers, along with cellulose nanocrystals [77,78]. The latter are discussed in more detail in the following section and, for the moment, results for the cellulose fibres only will be considered. In fact, these fibres were found to have only slight effects on the mechanical properties of the glass-ionomers (compressive strength, Young’s modulus, and diametral tensile strength) though, by contrast, biocompatibility was enhanced [79]. Fibres were added to the commercial conventional glass-ionomer Vidrion R (S.S. White, Brazil), a material with a reported compressive strength of only 49.15 MPa [78], which is too low for clinical use according to the relevant ISO standard [32]. Addition of cellulose fibres improved this and also enhanced both Young’s modulus and diametral tensile strength [78].

## 6. Nanoparticle Reinforcement

The influence of nanoparticles on the properties of glass-ionomers (mainly conventional) has been reported in some recent papers. For example, nanoparticle titanium dioxide has been incorporated into the commercial material Kavitan Plus (SpofaDental, Czech Republic) [80]. Following 24 h of storage in water, the flexural strength determined by three-point bending rose from 14 ± 3 MPa to 23 ± 3 MPa with 3 weight % addition of TiO_2_ [75]. However, using the same type of additive in the commercial material Ionofil Molar (VOCO, Germany) failed to produce any observable strengthening effect [81].

Other studies, however, have confirmed the reinforcing effect of nanoparticles [80,82,83]. A variety of nanoparticles have been used, such as titanium dioxide nanotubes [83] and nanoparticles [81,82], alumina nanoparticles [83], and zirconia nanoparticles [84]. Glass-ionomer cements with added nanoparticles have been found to be easier to mix than those without nanoparticles [85] and to contain fewer air voids or internal micro-cracks [86]. All of these features contribute to the observed increase in compressive strength.

One recent study used additions of TiO_2_, Al_2_O_3_ or ZrO_2_ nanoparticles to try to improve the strength of conventional glass-ionomers [85]. In addition to determining strength, the authors studied ion release by the cements. The cements used were two modern high-viscosity materials, Equia Fil (GC, Japan) and ChemFil Rock (Dentsply, Germany), with nanoparticles added at levels of 2, 5, and 10 weight %.

Results showed that the nanoparticles had varying effects, depending on type, amount, and storage time. Alumina significantly weakened Equia Fil at 24 h at all three loadings, whereas after 1 week, it weakened only the 5 weight % samples. In ChemFil Rock, for both the 1 day and 1 week storage times, it generally had no effect on strength, though at 5 weight % loading it caused a significant weakening. Findings were similarly mixed for zirconia nanoparticles, whereas titania nanoparticles generally increased strength, particularly at 10 weight % loading [85].

Studying the fracture surfaces by scanning electron microscopy confirmed that the addition of nanoparticles reduced the porosity, with no differences between the materials [86]. Ion release data showed that incorporating nanoparticles typically increased the levels of certain species released from the cement, mainly silica/silicate and phosphate. However, it did not cause release of the metal ions associated with the nanoparticles, i.e., Al, Ti, or Zr [86]. In all cases, no release occurred from the nanoparticle additives and, overall, the authors concluded that their addition to these high-viscosity cements was beneficial.

Other inorganic nanoparticles examined as additives for glass-ionomer cements have been hydroxyapatite [87] and fosterite [88]. The first of these was found to show enhanced antimicrobial activity against *Streptococcus mutans* bacteria, though no information was given on any changes in mechanical properties [87]. The latter was studied entirely for its effects on mechanical properties when added at levels in the range 1–4 weight %. Depending on the loading, this type of nanoparticle gave significant increases in compressive, flexural, and diametral tensile strength [88].

Organic nanoparticles have also been found to have positive effects on the properties of glass-ionomers, as shown by the cellulose nanocrystals previously mentioned [78,89]. These were found to have significant effects on the mechanical properties of a variety of commercial glass-ionomers (Maxxion, Vidrion R, Vitro Molar, Ketac Molar Easy Mix and Fuji Gold Label 9), raising the compressive strength by 10%, Young’s modulus by 61%, and the diametral tensile strength by 53% [78]. Fluoride release was also improved [88]. This was despite the fact that there appeared to be no chemical reaction between the cellulose nanocrystals and the cement matrix, as shown by the absence of any changes in the FTIR spectra [89]. Like the addition of cellulose microfibres, the nanocrystals enhanced the biocompatibility of the cements [79] and, overall, showed promise as additives for these cements.

Nanoparticulate silver has also been employed in experimental studies of conventional glass-ionomers [90,91]. The main reason for this has been that silver nanoparticles are capable of releasing Ag^2+^ ions for sustained periods of time, and these are extremely effective antibacterial species [92]. Silver ions have been found to show broad-spectrum antibacterial and antiviral properties at low concentrations as a result of their ability to disrupt bacterial cell walls and reduce the ability of the bacterial DNA to replicate [92]. Studies have confirmed that including silver nanoparticles in glass-ionomer cements leads to substantial improvements in their antibacterial properties [89]. As with nanoparticulate inorganic powders, compressive strength was improved when silver nanoparticles were present [90,91]. So, too, were micro-hardness and micro-shear strength to dentine [92]. Levels of addition have been low, between 0.1 and 0.5%, at which levels there is minimal discoloration of the cement.

## 7. Other Inorganic Powders

Inorganic powders with particle sizes bigger than the nanoscale have been added to glass-ionomers in attempts at reinforcement, but they have been uniformly unsuccessful [61]. An obvious candidate material is hydroxyapatite, which more or less corresponds to the mineral phase of the tooth and which is able to interact chemically with the polyacid component of the glass-ionomer cement [93]. An early study [94], using hydroxyapatite powder of unknown particle size at levels of 2.5, 5, 10, 20, and 25 weight % showed progressive reductions in compressive strength with increasing loading of additive in two experimental glass-ionomer cements.

In another study, the cement Fuji IX GP was used, and hydroxyapatite was added by substituting the glass powder at levels of 4, 12, and 28 volume % [95]. The mean particle size of the hydroxyapatite powder was 17 µm. Low amounts added made no significant difference to the strength, either compressive or diametral tensile, but higher levels resulted in substantial weakening. This confirmed the earlier findings.

Since these initial studies, there have been several other reports of the use of hydroxyapatite powders to reinforce various commercial glass-ionomer cements [96,97,98]. Fluorapatite has also been used [94], but in all cases, the addition of hydroxyapatite did not lead to reinforcement.

Other inorganic powders of reasonable particle size have been studied as possible reinforcing fillers. These include bioactive glass [99], montmorillonite clay [100], and borax [101]. In all cases, relatively low loadings were used, typically up to 10 weight % and often lower than that. Findings were consistent, with these additives causing increases in working/setting times but, unfortunately, reductions, often quite substantial, occur in compressive strength [99,100,101]. None of these powders has proven to be able to provide any reinforcement.

The question of reinforcement with larger particle inorganic powders has been complicated by glass-ionomer brands that the manufacturers claim are reinforced by the addition of ceramic powders, either of unspecified composition or zirconia [102,103]. These materials include “Amalgomer” (Advanced Healthcare Ltd., Tonbridge, UK) and “Zirconomer” (Shofu, Kyoto, Japan). Claims for them are considerable, with one group of authors describing them as “white amalgams”, a term which is completely unacceptable. Amalgams are, by definition, alloys of mercury [104], and this sort of inaccurate nomenclature should not be allowed to infiltrate the field of dental materials’ science.

The main issue with these materials is that there are no data to support the claim that they are reinforced. Some papers have appeared describing their properties [63,105,106,107], though most are in low-ranking journals of questionable provenance, and never reported with comparative data for control cements. One study, which is in a reputable journal, aimed to compare the properties of amalgam and “Amalgomer” using Hertzian indentation [102]. It showed that there were distinct differences between the materials, with “Amalgomer” being much weaker [103]. The authors, Wang and Darvell, described the “Amalgomer” material as an ordinary acid-base reaction GIC “but claimed to be reinforced with a ceramic powder” [107], showing that they had at least some skepticism about the claim.

This type of material is certainly strong, for example, “Amalgomer” has been reported to have a compressive strength of 323 MPa [63]. However, in the absence of information on the strength of the parent cement, there is nothing to support the claim of reinforcement. These materials are held to be suitable for use in the posterior dentition [103,105,106] but until better scientific data appear in more prestigious scientific journals, the case for this is unproven. Certainly, what information exists is not sufficiently compelling to alter the general conclusion that inorganic additives do not reinforce conventional glass-ionomer cements.

## 8. Conclusions

There have been many attempts to improve the strength of glass-ionomer cements over the years since they were first introduced to the dental profession in the mid-1970s. These have mainly been aimed at conventional glass-ionomers, though there are some reports involving resin-modified glass-ionomers. So far, very few of these approaches have proven to be successful. Good results have been obtained with certain fibres, notably hollow glass fibres, and with particular nanoparticles at specific loadings. In the latter case, the cement morphology is altered, with notable reductions in porosity. There are also commercial materials that claim to be reinforced with ceramics such as zirconia and, though these cements appear to be very strong, it is not clear that they are genuinely reinforced.

A major difficulty with work on this topic is that the literature contains a wide variety of mixing and testing conditions [61], despite the existence of relevant International Standards [32,33]. These standards fully specify test conditions in terms of specimen geometry, storage time, and conditions. In this way, they aim to eliminate wide variations in test regimes and the associated uncertainty in the results obtained; researchers really should use these defined test methods. Without this, their results are neither completely valid nor useful to the wider research community.

So far, none of the improvements in strength with certain additives have been translated into clinical materials, and existing commercial glass-ionomers should be used in posterior dentition with caution, paying careful attention to individual clinical conditions.

## Figures and Tables

**Table 1:** Mechanical properties of human tooth enamel and dentine.

Substance	Property	Value	Reference
Enamel	Hardness	2.0–3.5 GPa	[42]
	Young’s Modulus	80–120 GPa	[43,44,45]
	Fracture Toughness	0.67–3.93 MPa m^½^	[46]
Dentine	Hardness	0.3–0.7 GPa	[47]
	Young’s Modulus	10–40 GPa	[43,48,49]
	Fracture Toughness	1.1–2.3 MPa m^½^	[50,51]

**Table 2:** Load at failure of human teeth (*n* = 10) (standard deviations in parentheses).

Tooth Condition	Load at Failure/kg
Sound, uncut	104.65 (13.59)
Cavity prepared	48.88 (6.25)
Restored with composite layered obliquely	84.05 (14.03)

**Table 3:** Effect of 20 volume % reactive glass fibres in the strength of glass-ionomer cement.

Cement	Compressive Strength/MPa	Flexural Strength/MPa
Unreinforced	64	8.9
Fibre reinforced	170	15.6

**Table 4:** Results of incorporating hollow glass fibres into glass-ionomer cements (standard deviations in parentheses).

Material	Type	Compressive Strength/MPa	Flexural Strength/MPa	Diametral Tensile Strength/MPa
Fuji IX	Conventional	98.0 (12.0)	26.3 (9.0)	7.8 (1.7)
FIX/5% fibre		82.8 (13.8)	29.8 (6.0)	14.2 (2.6)
FIX/10% fibre		96.5 (9.0)	44.6 (4.0)	17.1 (3.5)
Fuji II LC	Resin-modified	123.0 (17.0)	55.2 (9.3)	17.7 (2.6)
FII/5% fibre		118.0 (8.3)	58.0 (8.4)	18.6 (4.2)
FII/10% fibre		154.2 (13.0)	75.2 (13.0)	23.0 (3.9)
